# Control of Flowering Time and Cold Response by a NAC-Domain Protein in *Arabidopsis*

**DOI:** 10.1371/journal.pone.0000642

**Published:** 2007-07-25

**Authors:** So Yeon Yoo, Yunhee Kim, Soo Young Kim, Jong Seob Lee, Ji Hoon Ahn

**Affiliations:** 1 Plant Signaling Network Research Center, School of Life Sciences and Biotechnology, Korea University, Seoul, Korea; 2 School of Biological Sciences, Seoul National University, Seoul, Korea; 3 Department of Molecular Biotechnology and Kumho Life Science Laboratory, College of Agriculture and Life Sciences, Chonnam National University, Gwangju, Korea; University of Chicago, United States of America

## Abstract

**Background:**

Plants must integrate complex signals from environmental and endogenous cues to fine-tune the timing of flowering. Low temperature is one of the most common environmental stresses that affect flowering time; however, molecular mechanisms underlying the cold temperature regulation of flowering time are not fully understood.

**Methodology/Principal Findings:**

We report the identification of a novel regulator, *LONG VEGETATIVE PHASE 1* (*LOV1*), that controls flowering time and cold response. An *Arabidopsis* mutant, *long* v *egetative phase 1-1D (lov1-1D*) showing the late-flowering phenotype, was isolated by activation tagging screening. Subsequent analyses demonstrated that the phenotype of the mutant resulted from the overexpression of a NAC-domain protein gene (*At2g02450*). Both gain- and loss-of-function alleles of *LOV1* affected flowering time predominantly under long-day but not short-day conditions, suggesting that *LOV1* may act within the photoperiod pathway. The expression of *CONSTANS (CO)*, a floral promoter, was affected by *LOV1* level, suggesting that *LOV1* controls flowering time by negatively regulating *CO* expression. The epistatic relationship between *CO* and *LOV1* was consistent with this proposed regulatory pathway. Physiological analyses to elucidate upstream signalling pathways revealed that *LOV1* regulates the cold response in plants. Loss of *LOV1* function resulted in hypersensitivity to cold temperature, whereas a gain-of-function allele conferred cold tolerance. The freezing tolerance was accompanied by upregulation of cold response genes, *COLD-REGULATED 15A* (*COR15A*) and *COLD INDUCED 1* (*KIN1*) without affecting expression of the C-repeat-binding factor/dehydration responsive element-binding factor 1 (*CBF/DREB1*) family of genes.

**Conclusions:**

Our study shows that *LOV1* functions as a floral repressor that negatively regulates *CO* expression under long-day conditions and acts as a common regulator of two intersecting pathways that regulate flowering time and the cold response, respectively. Our results suggest an overlapping pathway for controlling cold stress response and flowering time in plants.

## Introduction

Timing of the developmental transition to the reproductive phase is very important for plants to ensure successful reproduction and requires the proper perception and processing of a variety of stimuli. It is therefore not unexpected that the integration of complex signals from environmental and endogenous cues is necessary to enable plants to time this transition at the most advantageous moment. In *Arabidopsis*, at least four major floral promotion pathways are known to mediate signalling from the different cues: the photoperiod, vernalization, autonomous and gibberellin (GA) pathways [1]. Of these pathways, the photoperiod pathway plays an important role in controlling the timing of the developmental transition to flowering in *Arabidopsis*.

*CONSTANS* (*CO*) is an important floral promoter that acts within the photoperiod pathway [2]. It encodes a nuclear protein containing a CCT motif and two B-Box-type zinc-finger domains. The mRNA expression level of *CO* is modulated by the circadian clock and by day-length, and exposure to light is required to activate CO protein function [3], suggesting that *CO* fulfills the role of a mediator between the photoperiod/circadian clock and the floral integrators. Several upstream regulators of *CO* in photoperiod and circadian clock signalling have been identified. *GIGANTEA* (*GI*) positively mediates signalling from the circadian clock oscillators to *CO* [4]. *RED AND FAR-RED INSENSITIVE 2* (*RFI2*) [5], *CYCLING DOF FACTOR 1* (*CDF1*) [6], and *SUPPRESSOR OF PHYA-105 (SPA1)* [7] are light signalling molecules controlling *CO* expression downstream of photoreceptors. Although it has been firmly established that *CO* expression is regulated by the photoperiod and circadian clock, the regulation of *CO* expression by other environmental stimuli is poorly understood.

NAC (NAM, ATAF1, -2, and CUC2)-domain proteins are a class of transcription factors known to control multiple processes in plants, including developmental programs and abiotic/biotic stress responses [8]. One of the first reported *NAC* genes, *NO APICAL MERISTEM* (*NAM*) from petunia, plays a critical role in meristem formation [9]. As NAC-domain transcription factors are found only in plants, it is highly likely that they are involved in various plant-specific functions. As such, it is not surprising that NAC-domain genes comprise one of the largest transcription factor families in the compact *Arabidopsis* genome [10]. However, only a small number of the NAC-domain proteins have been studied to date [8], and the functions and regulation of most *Arabidopsis* NAC-domain genes are still largely unknown.

Temperature is an important environmental stimulus that affects flowering time, and an abundance of studies have indicated that it is highly probable that plants use different mechanisms to control flowering time in response to different temperature ranges. The thermosensory pathway genes play a role in regulating flowering in response to ambient growth temperature (moderately elevated or decreased temperatures) [11]–[13]. In response to near-freezing temperatures, plants exhibit cold acclimation or the vernalization response [14]. The duration of cold exposure to establish these respective responses is different, such that a few days of cold exposure is sufficient to initiate cold acclimation [15], whereas several weeks are required for vernalization [16]. It is generally accepted that cold acclimation is necessary for a plant to deal successfully with sudden temperature changes and that vernalization is required to ensure that flowering is inhibited until spring [14]. Although the molecular mechanisms involved in the control of flowering time by vernalization are well understood [14], [17], many questions have yet to be resolved in terms of cold temperature regulation of flowering time.

We report here a NAC-domain transcription factor, LOV1, which exerts its inhibitory effect on floral development by negatively regulating *CO* expression in a *GI*-independent manner. Mutations in *LOV1* led to altered responses to freezing temperatures. The loss-of-function *lov1* allele was hypersensitive to cold temperature, whereas a gain-of-function allele was tolerant to cold temperature. The freezing tolerance was attributed to the upregulation of cold response genes without altering expression of the C-repeat-binding factor/dehydration responsive element-binding factor 1 (*CBF/DREB1*) gene family. Based on our results, we propose that *LOV1* plays an important role in the coordination of cold response and flowering time.

## Methods

### Plant materials and growth conditions

All of the plants used in this study were *Arabidopsis thaliana* plants in the Columbia background, except for *co-2* and *gi-3*, which were in the L*er* background. *gi-2* plants harbouring *35S::GI* transgene were used for *GI* overexpressor plants (genotype: *35S::GI gi-2*). The *lov1-1D* mutant was isolated from an activation-tagged mutant library that had been generated in our laboratory [18]. A loss-of-function allele of *LOV1* that we used was a transposon insertion allele identified from the Exon Trapping Insert Consortium (EXOTIC) [19]. Since the allele was in the Landsberg background, we introgressed it four times into the Columbia background. The introgressed line was named *lov1*–*4*. Plants were grown in Sunshine Mix 5 (Quincy, Mich.) under long-day (16:8 h, light:dark) or short-day (8:16 h, light:dark) conditions at 23°C. The flowering time of the plants was measured by scoring the number of primary rosette and cauline leaves of at least 12 plants.

### Recombinant plasmid construction

A *LOV1* cDNA clone (C104984) was obtained from The Arabidopsis Information Resource (TAIR) and fused with the 35S promoter and *rbcS* terminator. The resulting construct, *pSYY004*, is referred to as *35S::LOV1* and was used for an overexpression analysis. The *35S::LOV1:GFP* (Green Fluorescence Protein) construct was generated by fusing a *LOV1* cDNA to *smGFP* [20]. To generate a *pLOV1*::*GUS* (β-glucuronidase) construct, we amplified a fragment of the *LOV1* promoter (–1,943 to –1, relative to a translational start) from the T16F16 BAC clone and cloned it into the pBI101.1 vector. The *pLOV1::LOV1:HA* transgene was generated by fusing the 1.9-kb *LOV1* promoter with HA-tagged *LOV1* cDNA in the pJHA212B vector [21].

### Expression analysis

RNA expression levels were measured by semi-quantitative reverse transcriptase (RT)-PCR followed by Southern hybridization [22]. Oligonucleotide sequences used to detect the mRNA of the genes studied are listed in Table S1. Seedlings for RNA extraction were harvested at the indicated Zeitgeber (ZT) times. Either the *UBIQUITIN10* (*UBQ10*) or *tubulin* gene was used as an internal positive control. For the diurnal expression analyses, plants were entrained under 12:12 h (light:dark) conditions for 10 days, then grown under continuous light conditions. The subcellular localization of *LOV1* was determined by using the *35S::LOV1:GFP* reporter gene. The *35S::LOV1:GFP* construct was introduced into onion (*Allium cepa* L.) epidermal cells by means of a particle gun (PDS-1000/He; Bio-Rad, Hercules, Calif.) using tungsten particles coated with plasmid DNA. The bombarded cells were then incubated at 22°C for 12 h, followed by staining with 4′-6-diamidino-2-phenylindole (DAPI; Sigma, St. Louis, Mo.); the GFP fluorescence was observed under a fluorescence microscope (model Axioskop 2 plus; Carl Zeiss, Germany) and photographed with AxioCam HRc (Carl Zeiss). For the histochemical GUS assay, whole seedlings were stained according to the procedure of Sessions *et al.* [23].

### Freezing-tolerance assay

*lov1*, *co-2,* and wild-type plant seeds were planted on soil and grown under long-day conditions. For the freezing treatment, the seedlings were placed in a controlled temperature chamber (CryMed® Freezer; ThermoForma, Marietta, Ohio) and subjected to freezing at –8°C for 2 h with or without cold acclimation. The plants were then transferred to a cold room (4°C) under white light and incubated overnight. Following thawing overnight, the plants were moved to a climate chamber maintained at 23°C and grown for 1 week under long-day conditions. For cold acclimation, 2.5-week-old seedlings were transferred to a cold room (4°C) and grown for 4 days prior to the freezing treatment.

## Results

### Isolation of *lov1-1D* mutants that show a late-flowering phenotype

**Figure 1:**
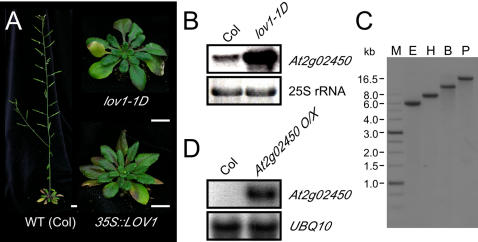
Characterization of gain-of-function alleles of *lov1*. (A) *lov1-1D* plants and *35S::LOV1* plants, an activation-tagged mutant and a cDNA overexpressor plant, respectively, grown under long-day conditions. These plants were germinated at the same time, and this photo was taken when a floral bud was seen in *35S::LOV1* plants. Bars = 1 cm (B) Expression of *At2g02450* in 18-day-old wild-type plants and *lov1-1D* mutants. 25S RNA was used as a loading control. (C) Genomic Southern blot analysis of *lov1-1D* mutants using *BAR* gene as a probe. B; *Bam*HI, E; *Eco*RI, H; *Hin*dIII, M; marker, P; *Pst*I (D) Confirmation of overexpression of *At2g02450* in 10-day-old transgenic plants (*At2g02450 O/X*) via northern blot analysis. *UBQ10* was used for a loading control.

A mutant that displayed delayed flowering under long-day conditions was isolated from an activation tagging library [18]. This mutant, which we denoted as *long vegetative phase 1-1D (lov1-1D),* flowered with 26.6 leaves under long days, whereas wild-type plants flowered with 15.5 leaves (Figure 1A). A plasmid rescue experiment [24] revealed that a T-DNA had been inserted into the last exon of *At2g02440* in chromosome 2. Despite the insertion of this T-DNA into the coding sequence of *At2g02440*, we concluded that the late-flowering phenotype was not associated with any disruption of the *At2g02440* gene structure because (1) the late-flowering phenotype was dominant and (2) *At2g02440* was a hypothetical protein whose cDNA was not detected in the known expressed sequence tag (EST) libraries. The next closest gene to the 35S enhancers in the T-DNA was *At2g02450*, which was located 8.9 kb from the right border of the T-DNA. Despite this long distance, the expression of *At2g02450* was significantly upregulated in *lov1-1D* mutants (Figure 1B). Genomic Southern hybridization using the *BAR* gene as a probe revealed a single T-DNA insertion in the *lov1-1D* mutants (Figure 1C). Taken together, these results strongly indicated that the late-flowering phenotype in *lov1-1D* plants was closely associated with the transcriptional activation of *At2g02450*.

We carried out a recapitulation experiment to confirm that overexpression of *At2g02450* caused the late-flowering phenotype. We expressed *At2g02450* cDNA under the control of the 35S promoter and chose transgenic lines that expressed *At2g02450* at high levels (Figure 1D). These transgenic plants showed late flowering (average leaf number: 23.7±5.1) under long-day conditions (Figure 1A), indicating that overexpression of *At2g02450* conferred the late-flowering phenotype observed in *lov1-1D* mutants. The overall phenotypes of the *lov1-1D* mutants and those of the transgenic plants that overexpressed *At2g02450* cDNA were similar. We therefore designated *At2g02450*, which encodes a NAC-domain transcription factor, as *LOV1*.

### Alteration of *LOV1* activity affects flowering time mainly under long-day conditions

**Figure 2:**
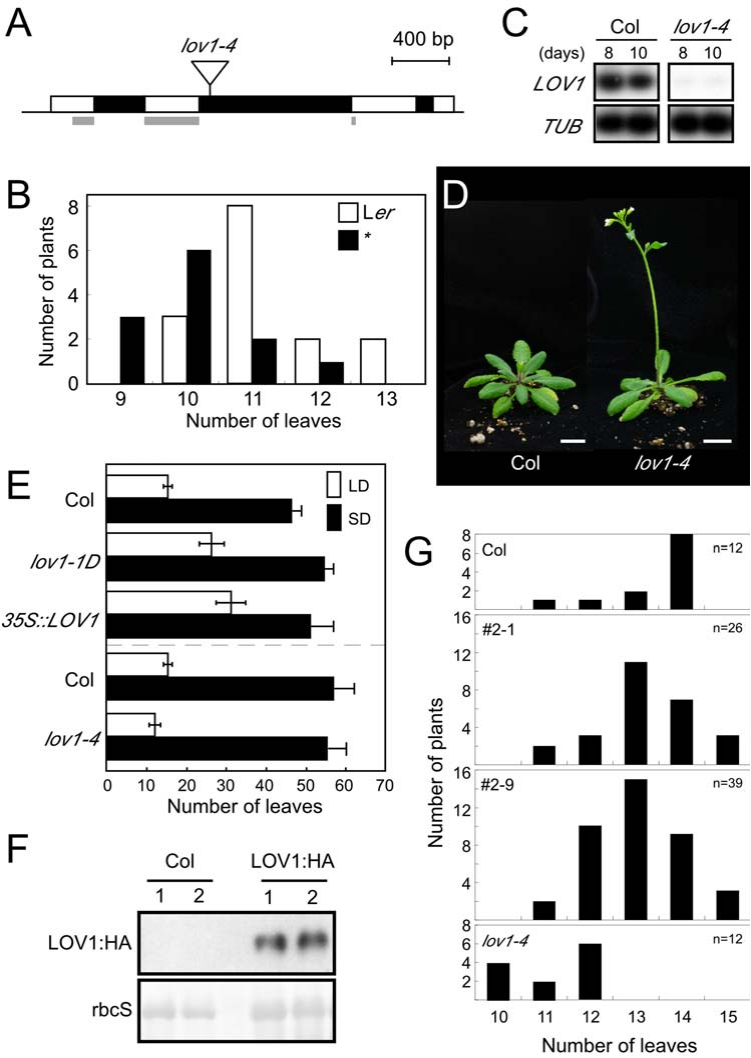
*lov1-4*, a loss-of-function allele of *LOV1*, and its flowering time. (A) Transposon (Ds) insertion map of *lov1-4* mutants. Open boxes and closed boxes indicate exons and introns of *LOV1,* respectively. Grey boxes mark a conserved NAC-domain. A transposon is denoted as a reverse triangle. (B) Distribution of the total number of leaves of the original transposon allele of *lov1-4* (asterisk) and wild-type L*er* plants. (C) Absence of *LOV1* expression in *lov1-4* mutants grown under long-day conditions. (D) Slight early flowering of *lov1-4* mutants under long-day conditions. Bars = 1 cm (E) Flowering time of *lov1-1D*, *35S::LOV1* and *lov1-4* plants under long-day and short-day conditions. Note that changes in the flowering times of these mutants were more prominent under long-day conditions. (F) Expression of the LOV1:HA proteins in two independent 10-day-old *pLOV1::LOV1:HA* transgenic plants. (G) Distribution of the total number of leaves of *lov1-4* plants with or without *pLOV1::LOV1:HA* transgene grown under long-day conditions. #2-1 and #2-9 are two independent lines of *pLOV1::LOV1:HA lov1-4* plants.

We identified a transposon allele in the Landsberg background which had a Ds transposon inserted into the second intron of *LOV1* (Figure 2A). Changes in flowering time in this line were very weak under long-day conditions (Figure 2B), possibly because Landsberg is a rapid-cycling accession. We introgressed this mutation into the Columbia background in order to facilitate its genetic analysis with other flowering time mutants that are in the Columbia background and then used this introgressed line (*lov1-4*) to investigate *LOV1* function. Semi-quantitative RT-PCR did not detect any *LOV1* expression in the mutants (Figure 2C), which suggested that *lov1-4* was most likely an RNA null allele.

Under long-day conditions, *lov1-4* plants flowered with 11.9±1.5 leaves (wild-type plants = 15.4±1.3 leaves), indicating that loss of *LOV1* function resulted in a slightly early-flowering phenotype (Figures 2D and 2E). However, no significant changes in flowering time were seen under short-day conditions. *35S::LOV1* plants and *lov1-1D* plants flowered with 51.5±6.0 and 55.1±3.0 leaves under short-day conditions (Figure 2E), respectively, whereas wild-type plants flowered with 48.1±3.0 leaves under the same conditions. In addition, the flowering time of *lov1-4* plants was also similar to that of the wild-type plants under short-day conditions (average leaf number: 55.7±4.6 vs. 57.3±5.0, respectively) (Figure 2E). These flowering time measurements indicated that both gain- and loss-of-function alleles of *LOV1* (hereinafter *lov1* mutants) exhibited altered flowering time under long-day conditions, but not under short-day conditions.

*35S::LOV1* and *lov1-4* plants resembled the wild-type plants in their response to GA treatment, vernalization, and different light qualities (Figure S1). These suggested that *LOV1* may not be involved in the genetic pathways that mediate these floral promoting signals. These data indicated that *LOV1* affected flowering time primarily under long-day conditions, which is characteristic of the photoperiod pathway mutants [25]. Taking into consideration the fact that the absence of *LOV1* function caused early flowering, our data suggest that *LOV1* may act as a floral repressor within the photoperiod pathway.

### Complementation analysis of *lov1-4* mutants

Since we introgressed *lov1-4* mutation into the Columbia background, it was still possible that the early flowering phenotype seen in the introgressed line resulted from a linked quantitative trait locus (QTL). To confirm that a mutation in *LOV1* caused the flowering time change in *lov1-4* mutants, we introduced the *LOV1* gene into *lov1-4* mutants by crossing *pLOV1::LOV1:HA* and *lov1-4* plants and then determined whether the early flowering defect was rescued. LOV1:HA protein expression in the transgenic plants was confirmed by means of Western blot analysis using HA antibodies (Figure 2F). *pLOV1::LOV1:HA lov1-4* plants flowered with 13.1 leaves under long-day conditions, whereas wild-type Columbia and *lov1-4* plants flowered with 13.4 and 11.2 leaves, respectively (Figure 2G). This indicated that LOV1:HA can functionally complement the *lov1-4* mutation and further suggested that the early flowering phenotype seen in *lov1-4* mutants resulted from the presence of the Ds transposon and, consequently, from the disruption of the *LOV1* gene structure.

### Expression patterns of *LOV1* in wild-type plants

**Figure 3:**
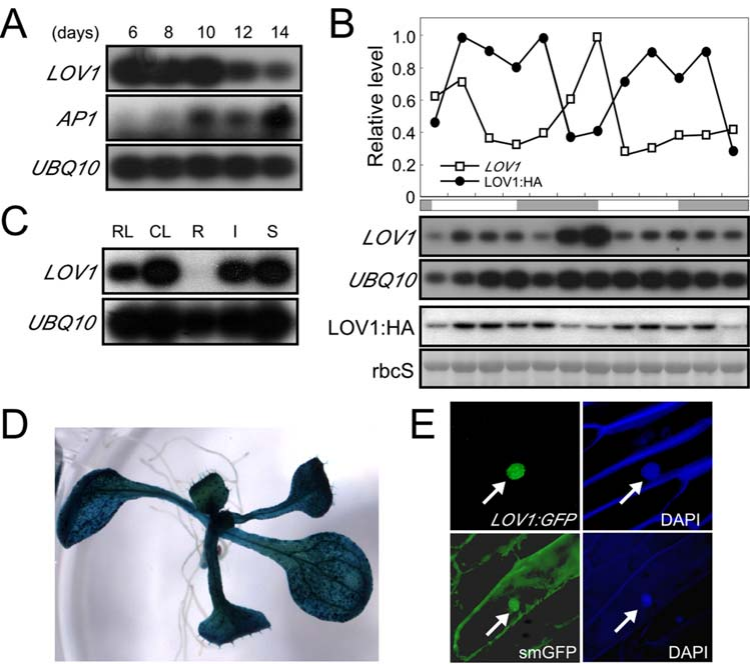
Expression pattern of *LOV1* in wild-type plants. (A) Time-course expression of *LOV1* and *AP1* in wild-type plants from day 6 to day 14 grown under long-day conditions. (B) Diurnal expression levels of *LOV1* transcripts and LOV1:HA fusion proteins. The expression level of the transcripts and LOV1:HA proteins was normalized against that of *UBQ10* and rbcS, respectively. The highest expression level was set to 1.0 for *LOV1* transcripts and LOV1:HA proteins. The rbcS is Ponceau S-stained blot used for a loading control. Open and grey boxes indicate subjective days and nights, respectively. (C) Tissue-specific expression pattern of *LOV1* in wild-type plants determined by RT-PCR. CL: cauline leaves; I: inflorescences; R: roots; RL: rosette leaves; S: stems (D) Histochemical GUS staining of 10-day-old *LOV1::GUS* transgenic plants. (E) Nuclear localization of LOV1:GFP protein. *35S::LOV1:GFP* was transiently expressed in onion epidermal cells and observed by fluorescence microscopy. smGFP was used as a control. DAPI was used to visualize the nucleus. An arrow indicates the nucleus.

A time-course analysis of *LOV1* expression in wild-type plants showed that *LOV1* was highly expressed in the early stages of seedling development but that its transcript levels subsequently gradually decreased (Figure 3A). The expression of *APETALA1* (*AP1*) [26], a molecular marker of floral transition, was also measured to identify the growth stages of the seedlings that we had harvested. *AP1* expression levels began to increase at day 10, suggesting that wild-type plants are in transition to flowering around this time point. This observation indicated that *LOV1* expression decreased during flower development, which is in good agreement with our proposal that *LOV1* acts as a floral repressor. We then measured circadian expression levels of *LOV1* to determine whether *LOV1* expression was controlled under the circadian clock [27]. The level of *LOV1* mRNA displayed a circadian oscillation with a peak at dawn under continuous light conditions (Figure 3B). Measuring the expression pattern of LOV1 protein using HA-tagged LOV1 protein, we found that the expression level of the LOV1-HA fusion protein also oscillated under continuous light conditions, with a broader peak around dusk and lower expression levels around dawn. These observations suggest that LOV1 protein began to accumulate after *LOV1* transcript levels decreased and that the mRNA and protein levels were regulated differentially. They also raised the possibility that LOV1 protein expression is under the control of the circadian clock or that *LOV1* function may be closely associated with circadian clock-controlled genes. An analysis of the tissue-specific expression patterns of *LOV1* in wild-type plants revealed that the *LOV1* transcript was detectable in all vegetative tissues except for the root (Figure 3C). This spatial expression pattern was also confirmed by the GUS reporter assay. *LOV1* promoter-driven *GUS* expression was detectable mainly in the above-ground parts of the seedlings (Figure 3D). A subcellular localization analysis of *LOV1* using LOV1:GFP protein revealed that *LOV1* is predominantly localized in the nucleus in transiently transformed onion epidermal cells, whereas free smGFP was detected throughout the cell, thereby suggesting that LOV1 is a nuclear protein (Figure 3E).

### Negative regulation of *CO* expression by *LOV1*

**Figure 4:**
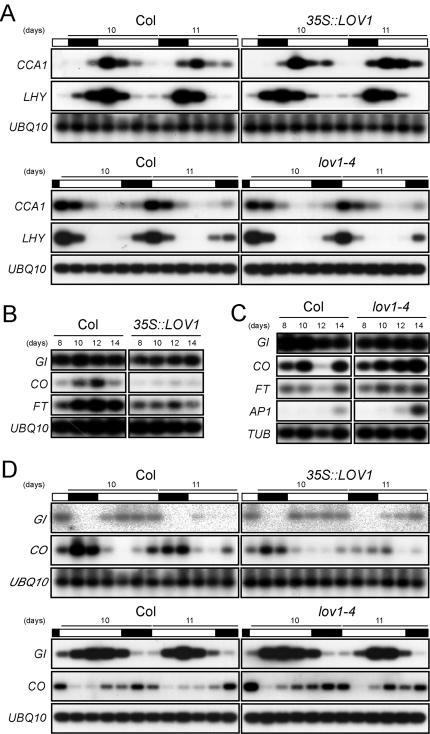
Expression patterns of the circadian clock and flowering time genes in wild-type plants and *lov1* mutants grown under long-day conditions. (A) Circadian rhythms of *CCA1* and *LHY* expression determined by RT-PCR analysis. Plants were harvested every 4 h for 48 h. Open and closed boxes indicate days and nights, respectively. (B) Time-course expression of *GI*, *CO*, and *FT* in *35S::LOV1* plants. Seedlings were harvested at ZT 10. (C) Time-course expression of *GI*, *CO*, *FT*, and *AP1* in *lov1-4* mutants. Seedlings were harvested at ZT 10. *AP1* was used as a molecular marker that indicates initiation of flower development. (D) Circadian expression of *CO* in *35S::LOV1* and *lov1-4* plants. Seedlings were harvested every 4 h for 48 h. *GI* and *UBQ10* were used as negative controls.

Based on our result that the main effect of *LOV1* on flowering time was seen under long-day conditions, we analyzed the expression levels of flowering time genes that act within the photoperiod pathway. Due to the oscillation of the transcript and protein levels of *LOV1*, we first monitored the expression of the circadian clock genes and found that circadian expression levels of *CIRCADIAN CLOCK ASSOCIATED 1 (CCA1)* [28] and *LONG HYPOCOTYL (LHY)* [29], the key regulators in the circadian clock function, were not altered by the overexpression of *LOV1* (Figure 4A). No significant differences in the peak patterns, period length, and amplitude of expression of these genes between *35S::LOV1* and wild-type plants were observed, although the peak expression of *LHY* appeared to shift slightly at day 10. Furthermore, the circadian oscillation of *CCA1* and *LHY* expression was not changed in *lov1-4* mutants. Therefore, it appeared that alterations in *LOV1* activity did not affect transcript levels of the circadian clock genes. This suggested that *LOV1* may act independently of the central oscillators of the circadian clock to regulate flowering time under long-day conditions.

An analysis of the time-course expression of genes acting downstream of the circadian clock showed that the expression level of *GI*, which may participate in a feedback loop of the plant circadian system [30], remained unaffected in *35S::LOV1* plants (Figure 4B). However, the expression levels of *CO*, which is an important floral promoter and known to act downstream of *GI* [4], were significantly downregulated in *35S::LOV1* plants (Figure 4B). Consistent with this result, the expression of *FT* [22], [31], a floral integrator gene acting downstream of *CO*, was also downregulated in *35S::LOV1* plants. These results indicated that downregulation of *CO* expression by *LOV1* overexpression may be the main regulatory factor explaining why *LOV1* overexpressor plants exhibited a late-flowering phenotype.

An expression analysis of the genes in the *lov1-4* mutants was conducted to confirm that *LOV1* affects the expressions of *CO* and *FT* but not *GI*. The result showed that *GI* expression was not altered in *lov1-4* mutants (Figure 4C). In contrast, *CO* expression was slightly upregulated and the expression of *FT* was also upregulated, which is consistent with the expression data obtained in *35S::LOV1* plants (Figure 4B). Furthermore, the expression of *AP1* [26] was precociously upregulated in *lov1-4* mutants (Figure 4C), which can be explained by the upregulation of *CO* and *FT* in the mutants.

Because *CO* expression levels oscillate in wild-type plants [3], we measured circadian expression levels of *CO* in *lov1* mutants to further confirm the negative regulation of *CO* expression by *LOV1*. The expression levels of *CO* were changed, such that overall expression levels of *CO* were lower in *35S::LOV1* plants but higher in *lov1-4* mutants (Figure 4D), although peak patterns and period length remained unaltered. It was notable that the peaks of *CO* expression were broader in *lov1-4* mutants. This observation suggested that more *CO* transcripts were present in *lov1-4* mutants under light conditions and that this increased level may subsequently activate *FT* expression and ultimately induce flowering. In contrast, *GI* transcript levels were largely unaffected by changes in *LOV1* activity. These data suggest that *LOV1* negatively regulates *CO* expression and that the de-repression of *CO* expression in the absence of *LOV1* function causes early flowering in *lov1-4* mutants.

### Genetic interaction studies of *LOV1*: overexpression of *CO* is epistatic to *LOV1* overexpression

**Figure 5:**
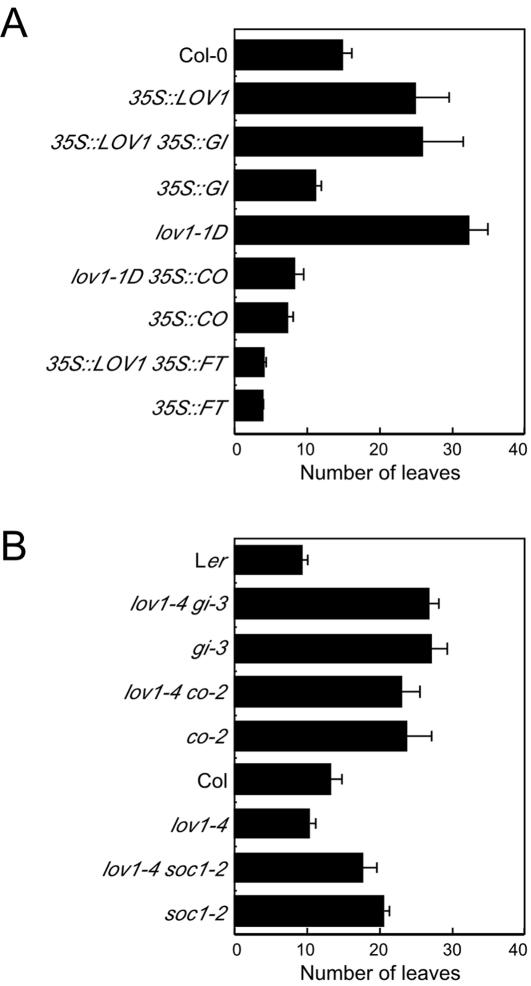
Genetic interaction of *LOV1* with genes that act within the photoperiod pathway. (A) Flowering time of the plants with *LOV1* gain-of-function alleles and mutants that double-overexpress *LOV1* and a flowering time gene under long-day conditions. (B) Flowering time of the plants with *LOV1* loss-of-function allele (*lov1-4* mutants) and double mutants under long-day conditions. Each error bar denotes the standard deviation.

To confirm the results obtained from the expression analysis, we investigated the genetic interaction between *LOV1* and genes that act downstream of the circadian clock in the photoperiod pathway by crossing gain- or loss-of-function alleles of *LOV1* with mutants in such genes. The result showed that the early-flowering phenotype of *35S::GI* plants was suppressed by *LOV1* overexpression (Figure 5A). Under long-day conditions, *35S*::*GI* plants flowered with 11.2±0.8 leaves, whereas plants overexpressing both *GI* and *LOV1* flowered late, with an average of 26.0 (±5.6) leaves, which was comparable to the late-flowering time of *35S::LOV1* plants (24.9±4.8 leaves). This result suggested that overexpression of *LOV1* masked the early-flowering phenotype of *35S*::*GI* and that *LOV1* may act downstream of *GI* or be independent of *GI*. *35S*::*CO* plants flowered with 7.2 leaves, whereas *lov1-1D* 35*S*::*CO* plants flowered with 8.4±1.2 leaves, indicating that increased *CO* activity was able to suppress the late-flowering phenotype of *35S::LOV1* plants. Consistent with this, the overexpression of *FT* was also epistatic to *LOV1* overexpression. The flowering of *FT* and *LOV1* double-overexpressor plants resembled that of *FT* single overexpressor plants (4.1±0.3 vs. 4.0±0.1 leaves, respectively). These data suggested that the late-flowering phenotype caused by the overexpression of *LOV1* was largely suppressed by the overexpression of *CO* or *FT*, but not by overexpression of *GI*. Thus, the results of the genetic analysis were consistent with the expression analysis (Figure 4), which further supports the hypothesis that *LOV1* acts upstream of *CO* and may act downstream or independent of *GI*.

Genetic crosses with *lov1-4* mutants were carried out to confirm the results of genetic studies performed with the gain-of-function alleles (Figure 5B). Both *gi-3 lov1-4* double mutants and *gi-3* single mutants showed a similar flowering time (average leaf number: 26.7±1.3 vs. 27.0±2.2, respectively), indicating that *lov1-4* mutation failed to suppress the late flowering of *gi-3* mutants and that de-repression of *CO* expression in the absence of *LOV1* function would not be sufficient to overcome the effect of *gi-3* mutations. This result suggested that *LOV1* may not be simply downstream of *GI*. *co-2 lov1-4* double mutants flowered similarly as did *co-2* single mutants (average leaf number: 22.8±2.6 vs. 23.6±3.4, respectively), indicating that *co-2* mutation largely suppressed early flowering of *lov1-4* mutants. This result is consistent with the epistatic relationship between *LOV1* and *CO. soc1-2 lov1-4* double mutants flowered with 17.6 leaves, whereas *lov1-4* and *soc1-2* single mutants flowered with 11.0 and 20.4 leaves, respectively, indicating that *soc1-2* mutation largely suppressed the early flowering of *lov1-4* mutants. Taking into consideration that *SOC1* is a direct downstream target of *CO* [32], this observation also supports the notion that *LOV1* acts upstream of *CO* and may act downstream or independently with *GI*.

### *lov1-4* plants are hypersensitive to freezing treatment

**Figure 6:**
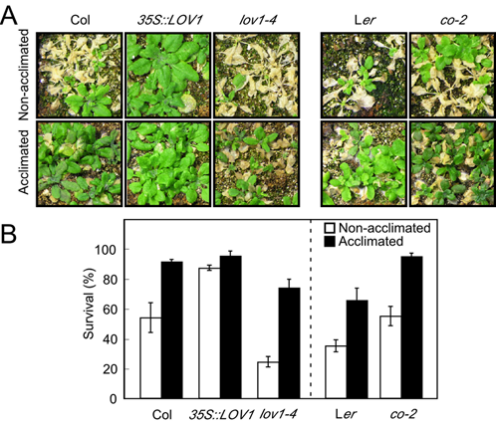
Altered sensitivity of *lov1* mutants to freezing temperature. (A) Freezing tolerance test of *lov1* mutants. 2.5-week-old *35S::LOV1* and *lov1-4* plants grown with or without cold acclimation were used for the freezing treatments. The photographs were taken 1 week after the freezing treatment. (B) Quantitative analysis of the plant survival rate 1 week after the freezing treatment.

In order to identify the upstream signalling pathway regulating LOV1 activity, the responses of *lov1* mutants to various stimuli, including hormones and abiotic stresses, were examined. *lov1* mutants displayed normal responses to phytohormones and most of the abiotic stresses (data not shown). However, *lov1-4* plants were hypersensitive to freezing temperature (–8°C), whereas *35S::LOV1* plants were tolerant (Figure 6A). Under our experimental conditions, 54% of the non-acclimated wild-type plants survived. In contrast, although only 24% of the *lov1-4* plants survived, the survival rate of *35S::LOV1* plants was 88%. When plants were cold-acclimated prior to the freezing treatment, the majority (92%) of wild-type plants became tolerant to the freezing temperature; in comparison, 74% of the *lov1-4* plants also became tolerant to the freezing treatment, suggesting that *lov1-4* plants are sensitive to freezing temperatures but are able to acclimate to the cold. Following a cold acclimation period, *35S::LOV1* plants were largely insensitive to the freezing treatment, and 95% of the plants survived the exposure to freezing at –8°C. These data suggested that *LOV1* may regulate the cold response in plants. As cold acclimation and constitutive freezing tolerance are under independent genetic control [33], [34], *LOV1* would appear to primarily control constitutive freezing tolerance in *Arabidopsis.*

Interestingly, we observed that a mutation in *CO* also led to freezing tolerance (Figure 6B). Of the *co-2* plants tested, 55% of the non-acclimated and 95% of the acclimated plants showed freezing tolerance; in comparison, 35% and 66% of the non-acclimated and acclimated wild-type L*er* plants showed freezing tolerance. Given our result that *LOV1* negatively regulates *CO* expression, this freezing-tolerant phenotype seen in *co-2* mutants is consistent with the phenotype of the *LOV1* overexpressor plants. Thus, our results suggest that both *LOV1* and *CO*, a well-known flowering time gene in the photoperiod pathway, may be involved in freezing tolerance.

### *LOV1* positively regulates *COR15A* and *KIN1* expression for the cold response

**Figure 7:**
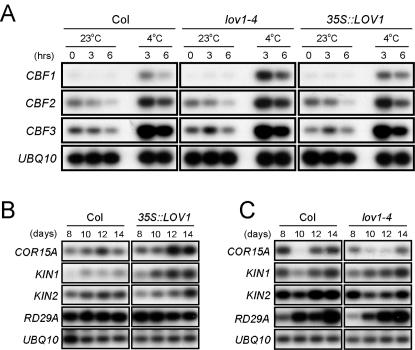
Expression analysis of cold response genes in *lov1* mutants grown under long-day conditions. (A) Expression patterns of *CBF/DREB1* genes in *lov1* mutants and wild-type plants. The plants were grown at 23°C for 10 days, and samples were harvested after 3 and 6 h with or without cold treatment (4°C). (B) Time-course expression of cold response genes in *35S::LOV1* and wild-type plants. (C) Time-course expression of cold response genes in *lov1-4* and wild-type plants.

To obtain empirical evidence of the mechanism by which *LOV1* affects cold response, we first determined the expression levels of *CBF/DREB1* family genes [35], the most important of the known transcription factors involved in the cold response, in *lov1* mutants. *CBF1* expression was not detectable at 23°C in wild-type plants, whereas *CBF2* and *CBF3* gene expressions were weakly detected (Figure 7A). When the wild-type plants were cold-treated at 4°C, the expressions of the *CBF/DREB1* genes were rapidly induced. These induction patterns of *CBF* genes at 4°C were also observed with *35S::LOV1* and *lov1-4* plants, indicating that *CBF* expression was not affected by *LOV1* expression.

We also monitored the expression of several cold-regulated genes in *lov1* mutants. We found that *COR15A* [36] and *KIN1* [37] expressions were upregulated in *35S::LOV1* plants, whereas the expressions levels of *RD29A* [38] and *KIN2* [39] were unaltered (Figure 7B). In *lov1-4* mutants, *COR15A* expression was downregulated (Figure 7C), which is consistent with the data obtained from *35S::LOV1* plants. However, *KIN1* expression levels remained unaltered in *lov1-4* mutants, suggesting that *LOV1* may not be essential for the expression of *KIN1*. These results suggest that the changed sensitivity of *lov1* mutants to cold temperature is, at least partially, conferred by the altered expression of *COR15A* and *KIN1* genes and may be regulated by means of one of several *CBF/DREB1*-independent signalling pathways in the cold response [40]. Considering that *CBF/DREB1* family genes do not have CRT/DRE motifs, a major *cis-*acting element in cold stress response [41], but that *COR15A* and *KIN1* do contain the motifs within their promoters, it is possible that LOV1 may bind to the motifs to regulate its target genes in the cold response.

## Discussion

An activation tagging screen resulted in the identification of *LOV1*, a member of the plant-specific NAC-domain transcription factors. Our data suggest that *LOV1* regulates flowering time by negatively regulating *CO* expression and that it also regulates the cold response by regulating the expressions of *COR15A* and *KIN1*. We propose that *LOV1* may play a pivotal role in coordinating flowering time and cold response.

Since *LOV1* negatively regulates *CO* expression, an important question is whether *LOV1* directly binds to a *cis*-acting element within the *CO* sequence. This possibility of such a binding is supported by the observation that the oscillation patterns of LOV1 protein levels and *CO* mRNA levels were largely in reverse phase to each other (Figures 2 and 3). However, an electrophoretic mobility shift assay that we performed did not identify the specific binding sites of LOV1 protein within the *CO* promoter sequence; rather, it appeared that LOV1 protein binds non-specifically to the promoter region of *CO* (S.Y.Y. and J.H.A., unpublished results). This suggests that LOV1 may not directly regulate *CO* expression and that *LOV1* may require an additional downstream gene that mediates signalling from *LOV1* to *CO*. However, we cannot exclude the possibility that non-specific binding to the *CO* promoter is required for changes in the chromatin structures of the *CO* locus, thereby leading to downregulation of *CO,* as seen in plant homeodomain finger proteins [42].

*LOV1* appears to control *CO* expression in a pathway that might be distinct from those of the photoperiod pathway and the circadian clock. *CO* is known to integrate the circadian clock and photoreceptor signalling processes in flower development [2]-[4], [43], [44]. In this study, however, we have shown that alterations in flowering time associated with changes in *LOV1* activity appear to be independent of *GI* and other circadian clock oscillators (Figure 4) and that *lov1* mutants did not show any significant changes in hypocotyl lengths under different light qualities (Figure S1). These results suggest that *LOV1* may function in a pathway that does not require the photoreceptor and circadian clock function. This concept is further supported by the observations that *LOV1*'s pattern of regulation of *CO* expression is different from those of previously identified regulators of *CO*, such as the *gi* mutation, which is epistatic to the *rfi2* mutation [5], the post-transcriptional regulation of *CO* by SPA1, which is likely short-day specific [7], [45], and CDF1, which directly binds to a *cis-*acting motif with the *CO* promoter [6]. Based on our results, *LOV1* is likely an additional upstream regulator that may mediate different a signalling pathway to *CO* (Figures 3 and 4). Given the fact that *CO* plays a central role in photoperiodic flowering and that the determination of the timing of flowering is critical for plants' successful reproduction, it is not particularly surprising that multiple independent regulators control the expression of *CO* in its function as a regulator of flowering.

One very interesting observation was that the downregulation of *CO*, a flowering time gene, also led to a tolerance to cold temperature, as this would suggest that *CO* could be an important regulator integrating developmental regulation and the cold stress response. The enhanced tolerance seen in *co* mutants is consistent with the finding that *35S::LOV1* plants are tolerant to cold temperature. Given our result that the overexpression of *LOV1* caused a decrease in *CO* transcript level, it is expected that *LOV1* overexpressor plants and a loss-of-function allele of *CO* exhibit a similar cold response (Figure 6). This implies that a subset of flowering time genes may play an additional role in the cold response. Consistent with this hypothesis is a recent finding that *GI*, an upstream regulator of *CO*, is involved in the cold stress response [46]. *GI* positively regulates tolerance to freezing temperature in a *CBF*-independent pathway, as observed in *lov1* mutants. Thus, it is probable that the canonical photoperiod pathway genes in flower development may play a dual role in flowering time control and the cold response. This may explain, at least partially, the delay or inhibition in the flowering of wild-type *Arabidopsis* plants that are exposed to intermittent cold stress.

Our proposal that *LOV1* may regulate the cold response is also supported by the observation that *lov1* mutants showed responses similar to those of *hos9* and *sfr6* mutants that are known to involve in cold signalling. *hos9* and *sfr6* mutants display altered sensitivities to freezing stress without affecting *CBF/DREB1* gene expression [47], [48], as also seen in *lov1* mutants. An interesting result is that *hos9* mutants also exhibit a flowering time phenotype [47], [48]; however, the precise mechanism by which *HOS9* regulates flowering time is unknown. In *sfr6* mutants, expressions of *KIN1* and *COR15A*, which contain the CRT/DRE motif in their promoters, were upregulated, as also seen in *35S::LOV1* plants. It is worth noting that *SFR6* may be a positive regulator of *LOV1* in the cold response since the level of *LOV1* transcripts was significantly decreased in *sfr6* mutants [49]. However, the *SFR6* gene has not yet been cloned. Future elucidation of the identity of *SFR6* will facilitate studies aimed at determining whether *SFR6* and *LOV1* act in the same pathway to control cold tolerance or whether they interact with each other to mediate the cold response.

In summary, our results suggest that the *LOV1* encodes a NAC-domain transcription factor that plays a pivotal function in flowering time regulation and cold response. *LOV1* acts as a floral repressor by negatively regulating the transcript level of *CO*, a central regulator of the photoperiod pathway. It also regulates the response of plants to freezing temperature by controlling a subset of cold response genes via a *CBF/DREB1*-independent pathway. Our results suggest a shared mechanism for controlling cold stress response and flowering time in plants and that the regulation of *CO* via *LOV1* may be common for these two distinct responses. Future investigations to determine the precise mechanism by which *LOV1* acts in two intersecting pathways will provide a better understanding of the modulation of reproductive development in plants under continuously changing temperature conditions.

## Supporting Information

Figure S1 Physiological responses in *lov1* mutants and wild-type plants. (A) Effect of vernalization on flowering time of *lov1* mutants. Hydrated seeds were treated with (+Ver) or without (-Ver) vernalization for 4 weeks at 4°C in a cold room under dark conditions. (B) Effect of GA treatment on flowering time of *lov1* mutants. Flowering time was measured under short-day conditions. 20 µM of GA was sprayed onto the entire aerial part of the plants until the floral bud was emerged. (C) Light effects on the elongation of the hypocotyls of *lov1* mutants. Note that the length of the hypocotyls was not affected by the quality of the monochromatic light.
(0.12 MB TIF)

Table S1 Oligonucleotides used for RT-PCR
(0.07 MB DOC)
