# Supplementary figures and images for: Control of Flowering Time and Cold Response by a NAC-Domain Protein in Arabidopsis
Source: PLoS One. 2007 Jul 25;2(7):e642. doi: 10.1371/journal.pone.0000642 (PMC1920552; doi:10.1371/journal.pone.0000642)

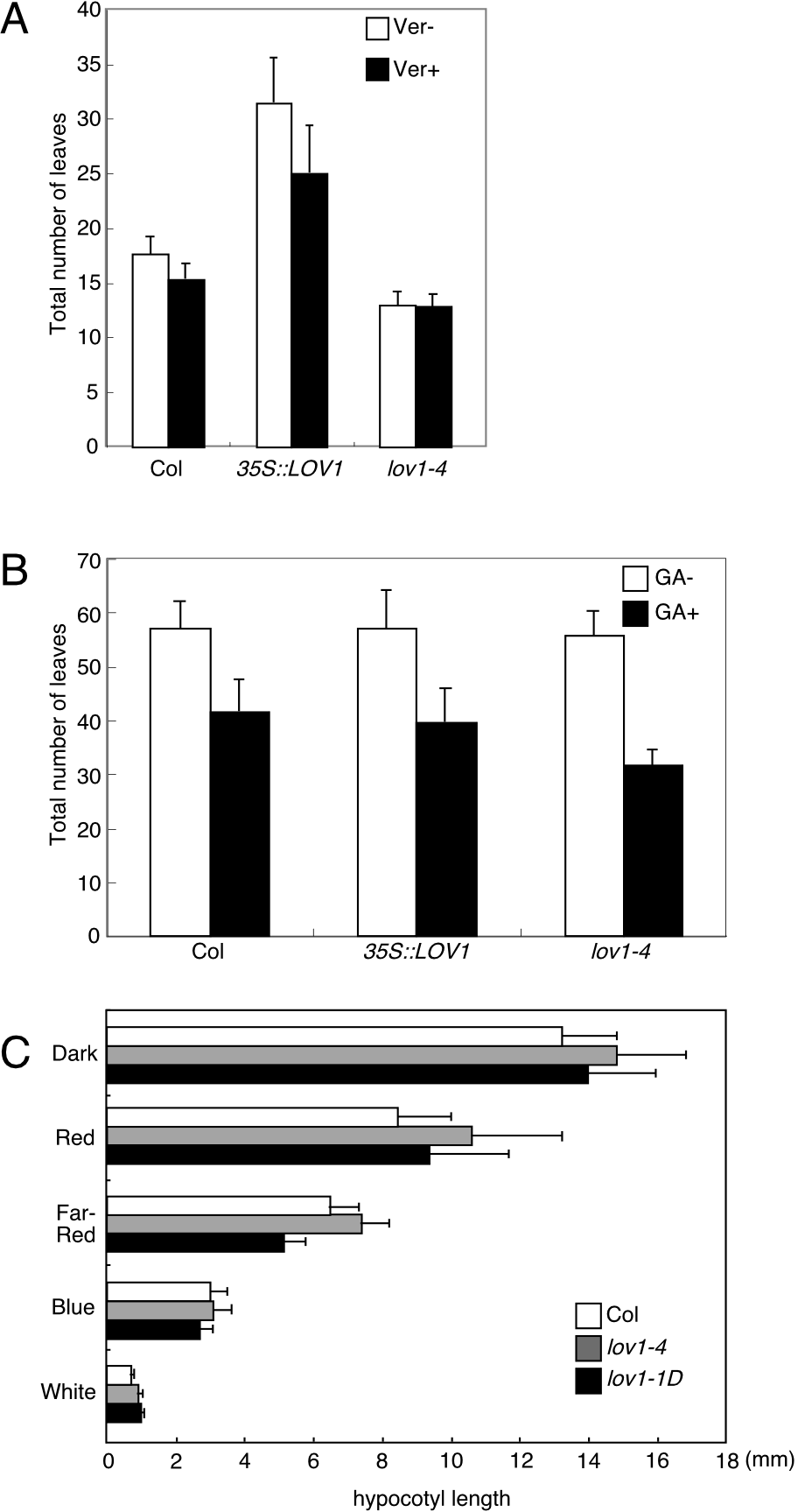

Supplement: Figure S1 — Physiological responses in lov1 mutants and wild-type plants. (A) Effect of vernalization on flowering time of lov1 mutants. Hydrated seeds were treated with (+Ver) or without (-Ver) vernalization for 4 weeks at 4°C in a cold room under dark conditions. (B) Effect of GA treatment on flowering time of lov1 mutants. Flowering time was measured under short-day conditions. 20 µM of GA was sprayed onto the entire aerial part of the plants until the floral bud was emerged. (C) Light effects on the elongation of the hypocotyls of lov1 mutants. Note that the length of the hypocotyls was not affected by the quality of the monochromatic light. (0.12 MB TIF) [file pone.0000642.s001.tif]
