# Supplementary material for: Control of Flowering Time and Cold Response by a NAC-Domain Protein in Arabidopsis
Source: PLoS One. 2007 Jul 25;2(7):e642. doi: 10.1371/journal.pone.0000642 (PMC1920552; doi:10.1371/journal.pone.0000642)
Supplement: Table S1 — Oligonucleotides used for RT-PCR (0.07 MB DOC) [file pone.0000642.s002.doc]

Table S1. Oligonucleotides used for RT-PCR

| **Gene** | **Primers** | **Sequences** |
| --- | --- | --- |
| *AP1* | FP1034 | 5’-TCCGACGTCAATACAAAC-3’ |
|  | FP1035 | 5’-GCACATCCGCACTAGAAAAAACCAAC-3’ |
| *CBF1* | CBF-F(All) | 5’-CAAACCGCTGAGATGGCAGCTCG-3’ |
|  | CBF1 R | 5’-CGATTTGTTTTGTATTGAATTAT-3’ |
| *CBF2* | CBF-F(All) | 5’-CAAACCGCTGAGATGGCAGCTCG-3’ |
|  | CBF2 R | 5’-AAACATTTGCATTTGACAACAAC-3’ |
| *CBF3* | CBF-F(All) | 5’-CAAACCGCTGAGATGGCAGCTCG-3’ |
|  | CBF3 R | 5’-ACTGAAACTGAATCAATTTAATT-3’ |
| *CCA1* | JH2389 | 5’-CGCAGTAGAATCAGCTCCAA-3’ |
|  | JH2390 | 5’-CCGATTCCAAGAAATCCTGT-3’ |
| *CDF1* | JH3704 | 5’-CGACGGTTTTAGAGGTTGCT-3’ |
|  | JH3705 | 5’-TCATCTCCGAGGCTGAAACT-3’ |
| *CO* | JH1015 | 5’-GCTAGACGCCATCAGCGAGTTCC-3’ |
|  | JH1016 | 5’-AAATGTATGCGTTATGGTTAATGG-3’ |
| *COR15* | COR15A-F | 5’-GGATGCCACAAACTAGGTCTTAC-3’ |
|  | COR15A-R | 5’-CATTAAAGAATGTGACGGTGACTGT-3’ |
| *FT* | JH1002 | 5’-ACTATAGGCATCATCACCGTTCGTTACTCG-3’ |
|  | JH1003 | 5’-ACAACTGGAACAACCTTTGGCAATG-3’ |
| *FKF1* | JH2527 | 5’-GTCTTCGAAGTCTTCACTGG-3’ |
|  | JH2528 | 5’-TTCCTCACACTCTCGTTCTT-3’ |
| *GI* | JH2428 | 5’-AAGCAGCAGCAGCAGTTGTC-3’ |
|  | JH2429 | 5’-GGGTGTGAAAGGCACCGTAT-3’ |
| *KIN1* | KIN1-F | 5’-CCTGAACAAGTAGCCGATTCGGGTCAAA-3’ |
|  | KIN1-R | 5’-GATAAAACATTATTTGAATATAAGTTTGGCT-3’ |
| *KIN2* | KIN2-F | 5’-TGAACAAGTAGCGATCCCAGTCAAC-3’ |
|  | KIN2-R | 5’-GAGGATGGTAAAACAAAGTTCTTAGAAC-3’ |
| *LHY* | JH2387 | 5’-CTTGGCTTCGAAATCTCCAG-3’ |
|  | JH2388 | 5’-CGAAAAGCTTTGAGGCAATC-3’ |
| *LOV1* | JH2158 | 5’-AAAGCCACCGGAGCTGATAG-3’ |
|  | JH2159 | 5’-CGGAATATTCGGTGGAGAGC-3’ |
| *RD29A* | JH3337 | 5’-GATAACGTTGGAGGAAGAGTCGGC-3’ |
|  | JH3338 | 5’-CAGCTCAGCTCCTGATTCACTACC-3’ |
| *SOC1* | JH1145 | 5’-GGATCGAGTCAGCACCAAACC-3’ |
|  | JH1146 | 5’-CCCAATGAACAATTGCGTCTC-3’ |
| *TUB* | JH3471 | 5’-CTCAAGAGGTTCTCAGCAGTA-3’ |
|  | JH3472 | 5’-TCACCTTCTTCATCCGCAGTT-3’ |
| *UBQ10* | JH1011 | 5’-GATCTTTGCCGGAAAACAATTGGAGGATGGT-3’ |
|  | JH1012 | 5’-CGACTTGTCATTAGAAAGAAAGAGATAACAGG-3’ |
